# Loss of ARID1A expression leads to sensitivity to ROS-inducing agent elesclomol in gynecologic cancer cells

**DOI:** 10.18632/oncotarget.10921

**Published:** 2016-07-29

**Authors:** Suet-Yan Kwan, Xuanjin Cheng, Yvonne T.M. Tsang, Jong-Sun Choi, Suet-Ying Kwan, Daisy I. Izaguirre, Hoi-Shan Kwan, David M. Gershenson, Kwong-Kwok Wong

**Affiliations:** ^1^ Department of Gynecologic Oncology and Reproductive Medicine, The University of Texas MD Anderson Cancer Center, Houston, TX, USA; ^2^ Cancer Biology Program, The University of Texas Graduate School of Biomedical Sciences at Houston, Houston, TX, USA; ^3^ School of Life Sciences, The Chinese University of Hong Kong, Shatin, New Territories, Hong Kong; ^4^ The Center for Anti-cancer Companion Diagnostics, Institutes of Entrepreneurial BioConvergence, Seoul National University, Gwanak-Gu, Seoul, Korea

**Keywords:** ARID1A, reactive oxygen species, elesclomol, ovarian cancer, drug sensitivity

## Abstract

Inactivating mutations in *ARID1A* are found in a broad spectrum of cancer types, with the highest frequency in gynecologic cancers. However, therapeutic strategies targeting *ARID1A*-mutant cancer cells remain limited. In this study, we aimed to identify drugs sensitivities in *ARID1A*-mutant cancer cell lines. By analyzing the Genomics of Drug Sensitivity in Cancer database, we found that *ARID1A*-mutant cancer cell lines were more sensitive to treatment with the reactive oxygen species (ROS)-inducing agent elesclomol. In a panel of 14 gynecologic cancer cell lines, treatment with elesclomol inhibited growth and induced apoptosis more potently in *ARID1A*-mutant cells. Knockdown of ARID1A in RMG1 and OVCA432 ovarian cancer cells resulted in increased sensitivity to elesclomol, whereas restoration of ARID1A expression in TOV21G ovarian cancer cells resulted in increased resistance to elesclomol. Furthermore, we found that knockdown of ARID1A expression resulted in increased intracellular ROS levels. In ovarian clear cell carcinoma patient samples, low expression of ARID1A correlated with high expression of 8-hydroxyguanosine, a marker for oxidative stress. In summary, we demonstrate for the first time that loss of ARID1A leads to accumulation of ROS and suggest that elesclomol may be used to target *ARID1A*-mutant gynecologic cancer cells.

## INTRODUCTION

Subunits of SWI/SNF are frequently inactivated in a variety of cancer types [1, 2]. In particular, AT-rich interactive domain-containing protein 1A (ARID1A) is the most frequently mutated SWI/SNF subunit in cancer [2], with the highest mutation frequency in gynecologic cancers, i.e. ovarian clear cell carcinomas, ovarian endometrioid carcinomas, and endometrial endometrioid carcinomas [3–7]. Recent studies demonstrated that ARID1A has tumor suppressive functions, such as regulation of epithelial-mesenchymal transition, p53 activity, the PI3K pathway, EZH2 targets, and DNA repair [8–13]. Although it has been demonstrated that ARID1A deficiency leads to sensitization to several inhibitors [10, 11, 13], therapeutic strategies that target *ARID1A*-mutant cancers remain limited.

Compared with normal cells, cancer cells have higher levels of intracellular reactive oxygen species (ROS) due to aberrant metabolic activity, oncogene activation, and the tumor microenvironment [14–17]. Although a moderate increase in ROS level can promote cell proliferation and survival [18–21], high ROS levels lead to activation of senescence and cell death [22–25]. To prevent intracellular levels of ROS from reaching toxic levels, cancer cells are dependent on an up-regulated antioxidant system and sensitive to further increases in ROS levels [14–16]. Several studies have demonstrated that treatment with agents that increase ROS can inhibit growth and induce apoptosis in cancer cells [26–29]. However, what predicts the sensitivity of cancer cells to ROS-inducing agents is not completely clear.

Previous studies demonstrated that SWI/SNF is required for oxidative stress resistance in different model organisms. In *Caenorhabditis elegans*, SWI/SNF is a co-factor for DAF-16 and required for DAF-16 functions, including longevity and resistance to oxidative stress [30]. In *Saccharomyces cerevisiae*, a genetic screen revealed that deletion of several SWI/SNF subunits resulted in increased sensitivity to oxidative stress [31]. Given that SWI/SNF is evolutionary conserved across several species, it is possible that SWI/SNF is also required for oxidative stress resistance in mammalian cells.

In this study, we sought to determine whether loss of ARID1A expression leads to increased sensitivity to a particular drug. We analyzed the Genomics of Drug Sensitivity in Cancer (GDSC) database [32] and found that *ARID1A*-mutant cancer cell lines were more sensitive to the ROS-inducing agent elesclomol than were *ARID1A*-wildtype cancer cell lines. Using gynecologic cancer cells, we validated that ARID1A deficiency led to increased sensitivity to treatment with elesclomol. Subsequently, we found that loss of ARID1A was associated with increased oxidative stress *in vitro* and in ovarian clear cell carcinoma patient samples. Taken together, our findings suggest that ARID1A protects cells against oxidative stress and ROS-inducing agents may be used to target *ARID1A*-mutant gynecologic cancer cells.

## RESULTS

### *ARID1A*-mutant cancer cell lines are more sensitive than *ARID1A*-wildtype cancer cell lines to treatment with the ROS-inducing agent elesclomol

To identify drug targets for *ARID1A*-mutant cancer cells, we analyzed the publicly available GDSC drug database [32] and compared the drug sensitivities of *ARID1A*-mutant and *ARID1A*-wildtype cancer cell lines. The GDSC database contains drug responses of more than 700 cancer cell lines of different cancer types to about 140 drugs. First, we determined the *ARID1A* mutation statuses of all the cancer cell lines using the Cancer Cell Line Encyclopedia database [33]. We excluded cell lines with no mutation or copy number alteration data from further analysis. We placed the remaining cell lines into *ARID1A*-wildtype (no detectable *ARID1A* mutations, n = 347) and *ARID1A*-mutant (*ARID1A* nonsense mutations, frameshift mutations, or deep deletions, n = 74) groups. We also excluded cell lines with *ARID1A* missense mutations, in-frame insertions/deletions, or splicing mutations from further analysis because the effect of these mutations on ARID1A protein expression and function is unclear.

The majority of drugs that exhibited significant differences (*P* < 0.05) in sensitivity between *ARID1A*-mutant and *ARID1A*-wildtype cancer cell lines were enriched in 1) inhibitors of the PI3K/AKT pathway (AZD8055, NVP-BEZ235, MK-2206, and GDC-0941) or 2) agents that induce DNA damage or inhibit the DNA damage response (cisplatin, KU-55933, and NU-7441) (Table 1). We noted that these enrichments are consistent with previously published data that ARID1A deficiency resulted in increased sensitivity to PI3K/AKT inhibitors [10] and agents that induce DNA double-strand breaks [12, 13].

**Table 1:** Drugs that exhibited significantly lower IC_50_ values in *ARID1A*-mutant cancer cell lines than in *ARDI1A*-wildtype cancer cell lines

Rank	Drug	Drug target	T-test	P-value	FDR (BH)
1	Elesclomol	Induced ROS accumulation	−5.0349	0.001996	0.1317
2	AZD8055	mTORC1/2	−4.9625	0.001996	0.1317
3	NVP-BEZ235	PI3K (class 1) and mTORC1/2	−3.4664	0.009980	0.2196
4	EHT 1864	Rac GTPases	−3.2140	0.003992	0.1756
5	MK-2206	AKT1/2	−2.9551	0.007984	0.2196
6	GW 441756	NTRK1	−2.9503	0.009980	0.2196
7	KU-55933	ATM	−2.8451	0.013970	0.2635
8	NU-7441	DNAPK	−2.6382	0.015970	0.2635
9	GDC0941	PI3K (class 1)	−2.6177	0.021960	0.3220
10	Cisplatin	DNA cross-linker	−2.2678	0.037920	0.4172
11	BIBW2992	EGFR and ERBB2	−2.2154	0.031940	0.3832

Abbreviations: FDR, false discovery rate; BH, Benjamini-Hochberg procedure.

Interestingly, we found that elesclomol, which potently induces ROS formation by disrupting the electron transport chain in the mitochondria [34], exhibited the greatest difference in sensitivity between the *ARID1A*-mutant and *ARID1A*-wildtype cancer cell lines (Table 1). As it has not been demonstrated that ARID1A is required to protect cells against oxidative stress in mammalian cells, we decided to focus on elesclomol for further validation.

### Elesclomol inhibits growth and induces apoptosis more potently in *ARID1A*-mutant than *ARID1A*-wildtype ovarian cancer lines

**Figure 1:**
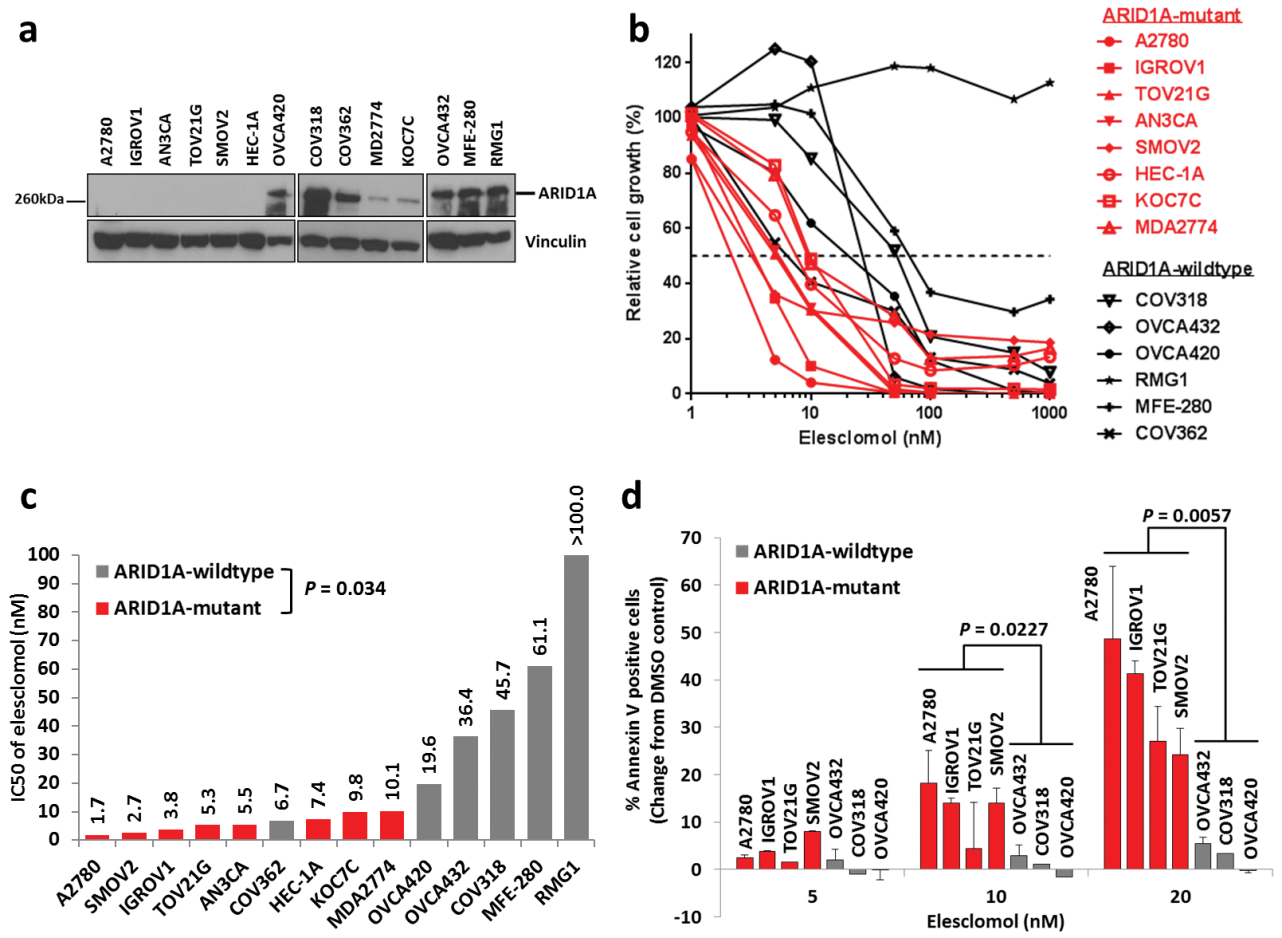
*ARID1A*-mutant cancer cell lines are more sensitive to treatment with the ROS-inducing agent elesclomol than *ARID1A*-wildtype cells **a.** Western blot analysis of ARID1A protein expression in a panel of 14 endometrial and ovarian cancer cell lines. **b.** Cell growth of endometrial and ovarian cancer cell lines treated with elesclomol for 72 h as measured using the WST-1 assay. Cell growth was quantified relative to DMSO treated controls. **c.** IC_50_ values of elesclomol in the cell lines in b. **d.** Apoptosis of *ARID1A*-mutant and *ARID1A*-wildtype cells treated with elesclomol for 72 h as measured using annexin-V and PI staining.

**Figure 2:**
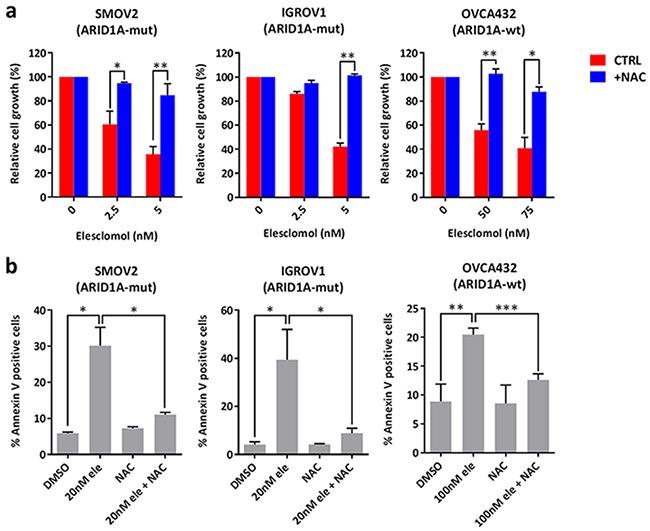
Treatment with elesclomol inhibits cancer cell growth and induces apoptosis by increasing ROS levels **a.** Cell growth of SMOV2, IGROV1, and OVCA432 ovarian cancer cells treated with elesclomol in the presence or absence of the antioxidant NAC for 72 h. Cell growth was measured using the WST-1 assay and quantified relative to DMSO treated controls. **b.** Apoptosis of SMOV2, IGROV1, and OVCA432 ovarian cancer cells treated with elesclomol (ele) for 72 h in the presence or absence of NAC for 72 h as measured using annexin-V and PI staining. *P < 0.05; **P < 0.01; ***P < 0.001.

To validate the findings from the GDSC database, we examined a panel of 14 ovarian and endometrial cancer cell lines. We first determined *ARID1A* mutation statuses in these cell lines using the Cancer Cell Line Encyclopedia database [33], DNA sequencing, and western blot analysis (Table 2 and Figure 1a). We found that the *ARID1A*-mutant cancer cell lines had significantly lower IC_50_s of elesclomol than did the *ARID1A*-wildtype cancer cell lines (*P* = 0.034) (Figures 1b and 1c). Interestingly, we noted that *ARID1A*-wildtype COV362 cells were very sensitive to treatment with elesclomol, which may be due to harboring a truncating mutation in BRCA1. A previous study has demonstrated that loss of BRCA1 results in increased ROS accumulation and sensitivity to oxidative stress in breast cancer cells [35]. Treatment with elesclomol at 10 and 20 nM also induced apoptosis more potently in *ARID1A*-mutant cancer cells than in *ARID1A*-wildtype cancer cells (*P* = 0.0227 and *P* = 0.0057, respectively) (Figure 1d). We confirmed that elesclomol exerted its effects through increasing ROS as addition of the antioxidant *N*-acetyl-L-cysteine (NAC) could abrogate the effects of elesclomol (Figure 2). Taken together, these results demonstrated that *ARID1A*-mutant cancer cell lines are more sensitive to treatment with elesclomol.

**Table 2:** *ARID1A* mutation statuses and ARID1A protein expression in a panel of ovarian and endometrial cancer cell lines

Cell line	Cancer type	*ARID1A mutation*	ARID1A protein expression
A2780	Ovarian	Q1430^*^^a^, R1721fs^a^	Absent
IGROV1	Ovarian	M274fs^a,b^, G1847fs^a,b^	Absent
AN3CA	Endometrial	G1848fs^a^	Absent
TOV21G	Ovarian	Q548fs^a,b^, N756fs^a,b^	Absent
SMOV2	Ovarian	G1740fs^b^	Absent
HEC-1A	Endometrial	Q404H^a^, Q1761C^a^, Q1835^*^^a^, Q2115^*^^a^	Absent
KOC7C	Ovarian	G276fs^b^, P1326fs^b^, A1517fs^b^	Absent
MDA2774	Ovarian	Q1947^*^^b^	Absent
COV362	Ovarian	Wild-type^a^	Present
OVCA420	Ovarian	ND	Present
COV318	Ovarian	Wild-type^a^	Present
OVCA432	Ovarian	ND	Present
MFE-280	Endometrial	Wild-type^a^	Present
RMG1	Ovarian	Wild-type^a,b^	Present

a Mutation status identified in the Cancer Cell Line Encyclopedia database.

b Mutation status identified in Sanger sequencing performed in this study. ND, not determined. Fs, frame-shift mutation.

* non-sense mutation.

### Knockdown of ARID1A expression increases the sensitivity of ovarian cancer cells to treatment with elesclomol

**Figure 3:**
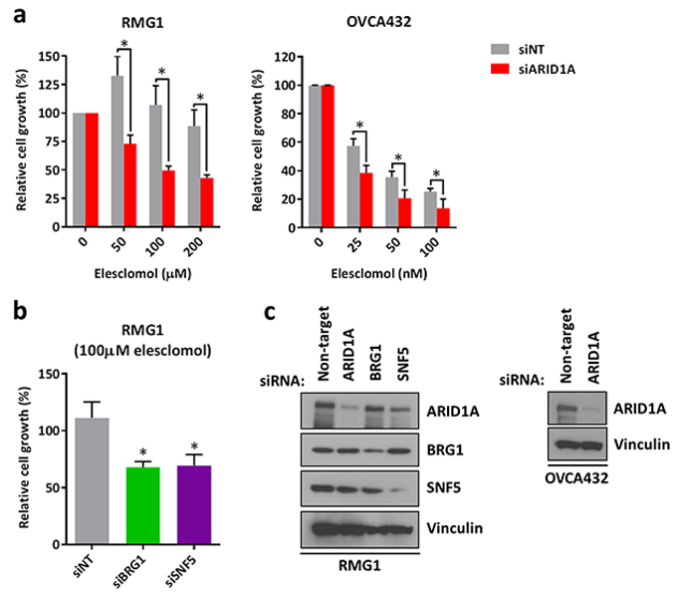
Knockdown of ARID1A expression in *ARID1A*-wildtype ovarian cancer cells results in increased sensitivity to treatment with elesclomol **a.** Cell growth of *ARID1A*-wildtype RMG1 and OVCA432 cells transfected with ARID1A and non-target siRNA for 24 h and treated with elesclomol for 72 h. **b.** Cell growth of RMG1 cells after transfection with BRG1, SNF5, and non-target siRNA and treatment as in a. **c.** Western blot analysis of RMG1 and OVCA432 cells after transfection with ARID1A, BRG1, SNF5, and non-target siRNA for 48 h. Cell growth was measured using the WST-1 assay and quantified relative to DMSO treated controls. *P < 0.05.

Next, we asked that whether loss of ARID1A expression is responsible for increased sensitivity to treatment with elesclomol. We found that depletion of ARID1A using siRNA in *ARID1A*-wildtype RMG1 and OVCA432 ovarian cancer cells resulted in increased sensitivity to elesclomol (Figure 3a). Although RMG1 cells were intrinsically highly resistant to treatment with elesclomol, depletion of ARID1A sensitized the cells to elesclomol in the micro-molar range (Figure 3a). In addition, we found that knockdown of the SWI/SNF core subunits BRG1 and SNF5 also resulted in increased sensitivity to elesclomol in RMG1 cells (Figure 3b). Down-regulation of ARID1A, BRG1, and SNF5 expression by siRNA were confirmed by western blot (Figure 3c).

To show that this effect was not limited to elesclomol, we also examined the sensitivity of these cells to treatment with another ROS-inducing agent, piperlongumine [27]. We found that ARID1A depletion in RMG1 cells also led to sensitization of the cells to piperlongumine (Supplementary Figure S1a and S1b). Similar to elesclomol, we found that piperlongumine inhibited growth by increasing ROS as treatment with NAC reversed the anti-proliferative effects of the drug (Supplementary Figure S1c).

### Re-expression of ARID1A increases the resistance of ovarian cancer cells to treatment with elesclomol

**Figure 4:**
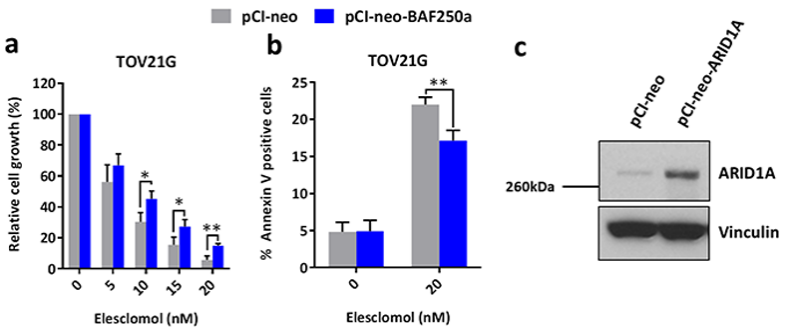
Re-expression of ARID1A in *ARID1A*-mutant ovarian cancer cells results in increased resistance to treatment with elesclomol **a.** Cell growth of *ARID1A*-mutant TOV21G cells after transfection with the pCI-neo-ARID1A and pCI-neo control vectors for 48 h and treatment with elesclomol for 72 h. Cell growth was measured using the WST-1 assay and quantified relative to DMSO treated controls. **b.** Apoptosis of TOV21G cells after transfection and treatment as described in **a** as measured using annexin-V and PI staining. **c.** Western blot analysis showing the re-expression of ARID1A in TOV21G cells. *P < 0.05; **P < 0.01.

To complement the siRNA experiments, we transiently re-expressed ARID1A in *ARID1A*-mutant TOV21G ovarian cancer cells and found that ARID1A re-expression resulted in increased resistance of the cells to treatment with elesclomol (Figure 4a and 4b). Western blot analysis confirmed that ARID1A was re-expressed in TOV21G cells after transfection with the pCI-neo-ARID1A vector (Figure 4c).

### Depletion of ARID1A leads to increased intracellular ROS level and cell proliferation

**Figure 5:**
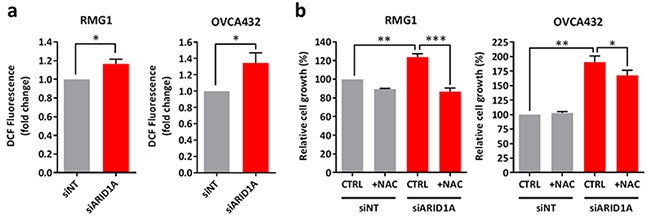
Knockdown of ARID1A expression in *ARID1A*-wildtype ovarian cancer cells results in increased intracellular ROS levels and cell growth **a.** Measurement of ROS levels in *ARID1A-*wildtype RMG1 and OVCA432 cells transfected with ARID1A and non-target siRNA for 72 h using DCFDA. **b.** Cell growth of RMG1 and OVCA432 cells after transfection with ARID1A and non-target siRNA for 24 h and treatment with the antioxidant NAC for 72 h. Cell growth was measured using the WST-1 assay and quantified relative to DMSO treated non-target control. *P < 0.05; **P < 0.01; ***P < 0.001.

Next, we asked that whether ARID1A affects intracellular ROS levels. We found that depletion of ARID1A resulted in an increase in intracellular ROS levels in RMG1 and OVCA432 cells by measuring 2′,7′-dichlorofluorescin diacetate (DCFDA) fluorescence (Figure 5a). We asked if the increase in intracellular ROS affects cell growth in ARID1A-knockdown cells. Upon ARID1A depletion, cell growth in RMG1 and OVCA432 cells was increased by 23% and 90% respectively (Figure 5b). Addition of NAC was able to fully inhibit the increase in cell growth upon ARID1A depletion in RMG1 cells and partially in OVCA432 cells (Figure 5b). These data suggests that up-regulation of ROS has growth promoting effects upon ARID1A depletion.

### Ovarian clear cell carcinoma patient samples with low expression of ARID1A display higher levels of oxidative stress

**Figure 6:**
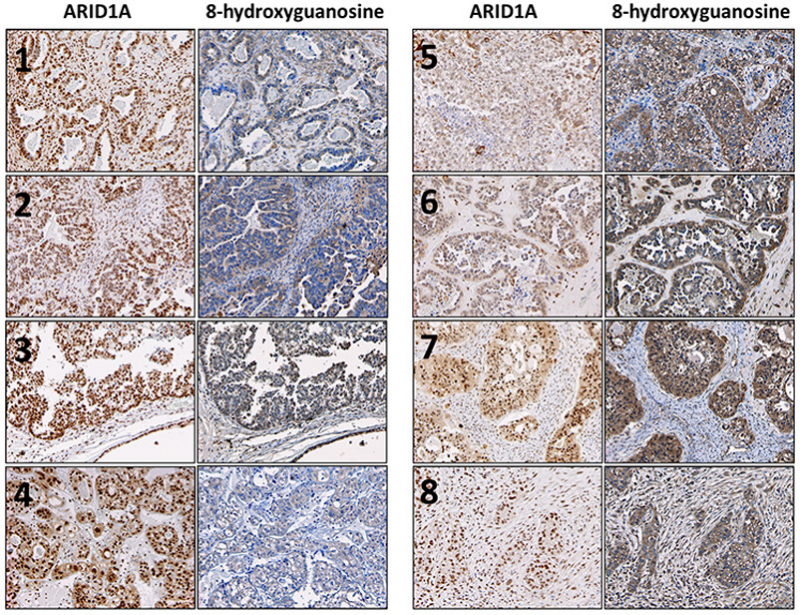
Ovarian clear cell carcinoma patient samples with low expression of ARID1A exhibit increased oxidative stress Expression of ARID1A and 8OHdG were determined using immunohistochemistry. Robust nuclear staining of ARID1A was observed for samples **1-4**. Robust cytoplasmic and nuclear staining of 8OHdG was observed for samples **5-8**. Photos were taken at 100×.

To demonstrate the biological significance of our *in vitro* findings, we examined the expression of 8-hydroxyguanosine (8OHdG) as a marker of oxidative stress in ovarian clear cell carcinoma patient samples. Representative images are shown in Figure 6. We found that samples with lower expression of ARID1A (Figure 6, samples 5-8) were associated with higher expression of 8OHdG compared to samples with higher expression of ARID1A (Figure 6, samples 1-4). Together with our *in vitro* data, these results show that loss of ARID1A is associated with increased oxidative stress.

## DISCUSSION

ROS has important roles in tumor development and progression [10, 14–16]. Although it has been demonstrated that ARID1A has important tumor suppressive functions in cancer [8–13], whether ARID1A regulates ROS has not been reported. In the present study, we demonstrate for the first time that loss of ARID1A leads to accumulation of ROS in gynecologic cancer cells. Consistent with our *in vitro* findings, we found that ovarian clear cell carcinoma patient samples with low ARID1A expression exhibited increased oxidative stress. We also found that the increase in ROS is fully required for growth promotion upon ARID1A depletion in RMG1 and partially required in OVCA432 cells. An increase in ROS levels can activate signaling pathways, transcription factors, and growth promotion [18–21, 36, 37], however, excessive ROS can cause oxidative damage to macromolecules and cell death [26, 38]. Therefore, we propose a model in which ARID1A loss leads to an increase in ROS that promotes cell growth, but renders the cells vulnerable to further oxidative stress. Further studies will be required to identify ROS-regulating genes that are affected by loss of ARID1A.

In our study, we also found that depletion of the SWI/SNF core subunits BRG1 and SNF5 also led to increased sensitivity to elesclomol in RMG1 cells, suggesting that other subunits of SWI/SNF are required for protection against oxidative stress. Previous studies also support this notion. In the Supplementary Data of the study of Du et al., knockdown of BRG1 led to increased sensitivity to H2O2 in lymphoblasts [39]. In *C. elegans*, SWSN-1, SWSN-3, and SWSN-8 (orthologs of human BAF155/170, BAF57, and ARID1A respectively) are co-factors of DAF-16 and required for DAF-16 mediated oxidative stress resistance [30]. A genetic screen in *S. cerevisiae* found that deletion of SNF2 (ortholog of human BRG1), SNF5, SNF6, and SWI3 (ortholog of human BAF155/170) resulted in increased sensitivity to oxidative stress [31]. As SWI/SNF subunits other than ARID1A are also frequently inactivated in cancer [1, 2], cancer cells with SWI/SNF mutations may be more sensitive to oxidative stress and ROS-inducing agents may be used to target these cancers.

Interestingly, previous studies have found that antioxidant activity is up-regulated in ovarian clear cell carcinomas. HNF1β, which is highly expressed in ovarian clear cell carcinomas, was found to reduce intracellular ROS levels and enhance oxidative stress resistance [40]. In addition, the NRF2 antioxidant pathway is activated in ovarian clear cell carcinomas, possibly due to mutations in the NRF2 negative regulator KEAP1 [41]. It is possible that up-regulation of the antioxidant system is required to combat the increase in ROS caused by loss of ARID1A. As up-regulation of antioxidant activity is associated with poor survival and resistance to chemotherapy [41–44], therefore, further understanding in how ARID1A regulates ROS levels and its possible co-operation with antioxidant pathways will be clinically relevant.

In summary, we found that loss of ARID1A leads to higher levels of ROS and sensitivity to the ROS-inducing agent elesclomol. Our study suggests a novel therapeutic strategy for *ARID1A*-mutant gynecologic cancer cells by inducing oxidative stress.

## MATERIALS AND METHODS

### Comparison of drug sensitivities in *ARID1A*-mutant cancer cell lines and *ARID1A*-wildtype cancer cell lines using the GDSC database

The drug sensitivities of cancer cell lines were downloaded from the GDSC database (release 4, March 2013) [32]. Mutation statuses and copy numbers of ARID1A in cancer cell lines were obtained from the Cancer Cell Line Encyclopedia database using the cBioPortal for Cancer Genomics (www.cbioportal.org) [33]. Cell lines with no mutation or copy number alteration data were excluded from further analysis. Based on *ARID1A* mutational status, cell lines were placed into *ARID1A*-wildtype (no detectable *ARID1A* mutations, n = 347) or *ARID1A*-mutant *(ARID1A* nonsense mutations, frameshift mutations, or deep deletions, n = 74) groups. In addition, cells lines with missense mutations, in-frame insertions/deletions, or splicing mutations in *ARID1A* were excluded from further analysis because the effect of these mutations ARID1A expression and function are unclear.

To detect differences in drug sensitivity between *ARID1A*-mutant and *ARID1A*-wildtype cell lines, permutation tests were performed using the marker selection function of the GENE-E matrix visualization and analysis platform (www.broadinstitute.org/cancer/software/GENE-E/). For each drug, a test statistic was calculated to assess the difference in drug response between *ARID1A*-mutant and *ARID1A*-wildtype cell lines. Next, the significance of the test statistic score was estimated in 1000 permutations. Multiple hypothesis testing was corrected by computing both the false discovery rate and the family-wise error rate. A two-tailed *t*-test was used to calculate significance. A negative t-test score plus a significant *P*-value suggested that *ARID1A*-mutant cancer cell lines were more sensitive to the corresponding drug than *ARID1A*-wildtype cancer cell lines.

### Cell culture

All cell lines were cultured in RPMI-1640 medium, supplemented with 10% FBS and 1% penicillin/streptomycin unless otherwise stated. RMG1 and TOV21G ovarian cancer cells and HEC-1A endometrial cancer cells were purchased from the American Type Culture Collection (Manassas, VA, USA). HEC-1A cells were cultured in McCoy's 5A medium. MDA2774 ovarian cancer cells were a gift from Dr. Ralph Freedman (The University of Texas MD Anderson Cancer Center). OVCA420 and OVCA432 ovarian cancer cells were gifts from Dr. Robert Bast (MD Anderson). AN3CA endometrial cancer cells were purchased from the MD Anderson Characterized Cell Line Core and cultured in Eagle's minimum essential medium. A2780, COV318, and COV362 ovarian cancer cells and MFE-280 endometrial cancer cells were purchased from the European Collection of Cell Cultures. COV318 and COV362 cells were cultured in Dulbecco's modified Eagle's medium supplemented with 2 mM L-glutamine. MFE-280 cells were cultured in 40% RPMI-1640 medium and 40% minimum essential medium (with Earle's salts) supplemented with 2 mM L-glutamine, 20% FBS, and 1× insulin-transferrin-sodium selenite. SMOV2 and KOC7C ovarian cancer cells were gifts from Dr. Hiroaki Itamochi (Tottori University, Tottori City, Japan). IGROV1 ovarian cancer cells were a gift from Dr. Susan Holbeck (National Cancer Institute). All cell lines were cultured at 37 °C in 5% CO_2_ and were tested negative for mycoplasma. The cell lines were maintained for 20-30 passages.

### PCR amplification of ARID1A

Genomic DNA was harvested from cells using the PureLink Genomic DNA mini kit (Life Technologies, Carlsbad, CA, USA) following the manufacturer's protocol. The sequences of PCR primers and PCR cycling conditions used to amplify exons 1-20 of ARID1A were previously described [4]. PCR was performed in 50 μL reactions containing MyTaq Red Mix (Bioline, Taunton, MA, USA), 1.5 μM of forward primer, 1.5 μM of reverse primer, 6% DMSO and 20 ng of DNA. PCR reactions were purified using the PureLink PCR purification kit (Life Technologies). Purified PCR products were sent to the MD Anderson Sequencing and Microarray Core for Sanger sequencing.

### Western blot analysis

Cells were washed twice in ice-cold PBS and scraped on ice in ice-cold RIPA buffer (Sigma-Aldrich, St. Louis, MO, USA) supplemented with protease inhibitor cocktail (Sigma-Aldrich). Protein lysates were collected after centrifuging the cells at 13,000 rpm for 10 min at 4 °C. For each sample, 25 μg of protein was loaded onto a SDS-PAGE gel. After transferring the protein to a nitrocellulose membrane, the membrane was incubated with primary antibodies against ARID1A (Sigma-Aldrich), BRG1 (Cell Signaling Technology, Danvers, MA, USA), SNF5 (Cell Signaling Technology), and vinculin (Cell Signaling Technology). Then, the membrane was incubated with anti-rabbit horseradish peroxidase-conjugated secondary antibody (Cell Signaling Technology). The bands on the membrane were visualized using enhanced chemiluminescence plus western blotting reagent (Amersham Biosciences, Little Chalfont, UK).

### Chemicals

Elesclomol and piperlongumine were purchased from Selleck Chemicals (Houston, TX, USA) and reconstituted in DMSO. NAC was purchased from Sigma-Aldrich and reconstituted in dH_2_O. Working solutions were made fresh before each experiment.

### Cell growth assays

Cells were plated in 96-well plates for 24 h before the addition of drugs. After 72 h of treatment with the drugs, cell growth was measured using WST-1 reagent (Roche, Indianapolis, IN, USA) according to the manufacturer's protocol. Dose-response curves were constructed using the Prism software program (version 6, GraphPad Software, La Jolla, CA, USA) and IC_50_ values were interpolated from the graphs.

### Annexin V staining

Cells were treated with the indicated drugs for 72 h and collected by centrifugation at 1,000 rpm for 5 min at 4 °C. The cells were washed in ice-cold PBS and resuspended in annexin binding buffer (10 mM HEPES, 140 mM NaCl, 2.5 mM CaCl_2_, pH 7.4; Life Technologies). For each sample, 1 × 10^5^ cells were stained in 100 μL of annexin binding buffer with 5 μL of annexin V-APC (BD Pharmingen, San Diego, CA, USA) for 15 min at room temperature in the dark. Prior to analysis, 400 μL of annexin binding buffer and 100 ng/mL propidium iodide (BD pharmingen) were added to each sample. For each sample, at least 10,000 cells were analyzed using a Gallios Flow Cytometer (Beckman Coulter, Brea, CA, USA). Cells were gated to include single cells only. Data analysis was performed using the Kaluza Analysis software (version 1.3, Beckman Coulter). The annexin V-positive cells included both annexin V/PI double-positive and annexin V-positive/PI-negative cell populations.

### siRNA transfection

Cells were transfected with 20 nM siGENOME SMARTpool ARID1A, SNF5, and BRG1 siRNAs (Dharmacon, Lafayette, CO, USA) and Lipofectamine RNAiMAX (Life Technologies) according to the manufacturer's protocol. Control cells were transfected with siGENOME non-targeting siRNA pool #2 (Dharmacon). For cell growth assays, cells were transfected for 24 h and then treated with the indicated drugs for 72 h.

### Re-expression of ARID1A

The pCI-neo-ARID1A vector was a gift from Dr. Weidong Wang (National Institutes of Health) and has been described previously [45]. Due to several non-synonymous mutations in the ARID1A open reading frame in the original vector, mutagenesis was carried out in the Custom Cloning Core at Emory University to restore the ARID1A open reading frame to the wild-type sequence. Cells were plated in a 6-well plate and transfected with 2 μg of pCI-neo-ARID1A vector and Lipofectamine 3000 (Life Technologies) according to the manufacturer's instructions. Control cells were transfected with empty pCI-neo vector. Twenty-four hours after transfection, cells were trypsinized and re-plated for subsequent experiments.

### Cellular ROS assay

Cells were harvested by trypzinisation and washed with ice-cold PBS. For each sample, 5 × 10^5^ cells were stained with 10 μM DCFDA (Sigma-Aldrich) in 1 mL of PBS and incubated for 30 min at 37 °C in the dark. The cells were gently mixed every 10 min to prevent them from setting at the bottom. Stained cells were collected by centrifugation and resuspended in 500 μL of PBS. Prior to analysis, 2 μg/mL of DAPI (Life Technologies) was added to each sample. For each sample, at least 20,000 cells were analyzed using a Gallios Flow Cytometer and the cells were gated to include live and single cells only. The Kaluza Anlaysis Software was used to analyze the mean fluorescence.

### Immunohistochemistry staining

Paraffin-embedded sections from patients with ovarian clear cell carcinomas were obtained from the archives of the Department of Pathology at The University of Texas MD Anderson Cancer Center. All cases were reviewed and confirmed as ovarian clear cell carcinomas by a gynecologic pathologist (J.S.C.). All tissue specimens were collected and archived previously under protocols approved by the institutional review board. Immunohistochemistry staining of the sections was performed as previously described [46]. Slides were stained with anti-8OHdG (1:200, EMD Millipore) and anti-ARID1A antibodies (1:100, Sigma-Aldrich).

### Statistical analysis

Values are presented as the means and error bars represented the standard deviation. Unless otherwise stated, *P*-values were determined using the Student *t*-test. *P*-values of <0.05 were considered significant.

## SUPPLEMENTARY MATERIALS FIGURE


