# Supplementary material for: Loss of ARID1A expression leads to sensitivity to ROS-inducing agent elesclomol in gynecologic cancer cells
Source: Oncotarget. 2016 Jul 29;7(35):56933–43. doi: 10.18632/oncotarget.10921 (PMC5302963; doi:10.18632/oncotarget.10921)
Supplement: Supplementary file 1 [file oncotarget-07-56933-s001.pdf]

Loss of ARID1A expression leads to sensitivity to ROS-inducing agent elesclomol in gynecologic cancer cells

SUPPLEMENTARY FIGURE

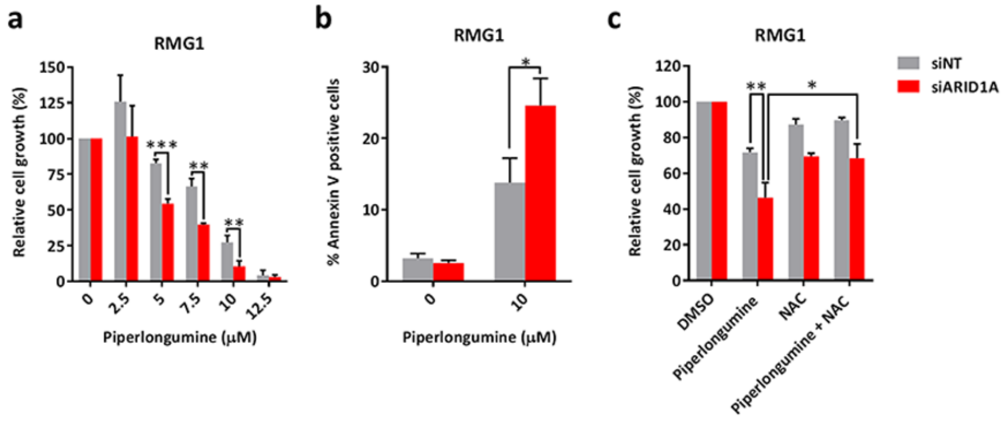

Supplementary Figure S1: Knockdown of ARID1A expression in *ARID1A*-wildtype ovarian cancer cells results in increased sensitivity to treatment with the ROS-inducing agent piperlongumine. a. Cell growth of *ARID1A*-wildtype RMG1 cells transfected with ARID1A and non-target siRNA for 24 h and treated with piperlongumine for 72 h. **b.** Apoptosis of RMG1 cells after transfection and treatment as described in **a** as measured using annexin-V and PI staining. **c.** Cell growth of RMG1 cells transfected and treated with 5 μM of piperlongumine as described in **a,** but in the presence or absence of the antioxidant NAC. Cell growth was measured using the WST-1 assay and quantified relative to DMSO treated non-target control. $^*P < 0.05$; $^{**}P < 0.01$; $^{***}P < 0.001$.
